# Short-term clinical and radiographic outcomes of arthroscopic-assisted uni-portal spinal surgery discectomy with versus without ligamentum flavum preservation: a retrospective comparative cohort study

**DOI:** 10.3389/fsurg.2026.1964665

**Published:** 2026-10-23

**Authors:** Yuan Jiang, Shijie Fan, Tianyu Qin, Haoxiang Chen, Bo Wang, Feng Hu, Jialin Chen, Yumeng Du, Jie He, Houqing Long, Wei Guo

**Affiliations:** 1 Department of Spine Surgery, Changde Hospital, Xiangya School of Medicine, Central South University (The First People’s Hospital of Changde City), Changde, China; 2 Division of Spine, Department of Orthopedics, Shenzhen People’s Hospital (The First Affiliated Hospital, Southern University of Science and Technology; The Second Clinical Medical College, Jinan University), Shenzhen, China

**Keywords:** AUSS endoscope, clinical outcomes, ligamentum flavum, lumbar disc herniation, lumbar discectomy

## Abstract

**Background:**

To compare ligamentum flavum preservation vs. resection during AUSS for single-level lumbar disc herniation and evaluate the clinical value of preservation in this setting.

**Methods:**

This retrospective comparative cohort study included 65 patients (Sept 2024–Sept 2025): Group A (preservation, *n* = 30) and Group B (resection, *n* = 35). Both underwent AUSS-assisted nucleotomy. Outcomes included operative parameters, complications, VAS for low back and leg pain, ODI, modified MacNab criteria, and imaging parameters (spinal canal cross-sectional area and sagittal and transverse diameters). Assessments were VAS preoperatively and at day 2; ODI preoperatively and at day 2, 1 month, and 3 months; MacNab at 3 months; disc height preoperatively and at 3 months; and CT preoperatively and at 3 and 6 months (65/65/65).

**Results:**

Operative time, blood loss, and hospital stay did not differ significantly (*P* > 0.05). Both groups showed significant postoperative improvements in VAS and ODI vs. baseline (*P* < 0.05), with no intergroup differences (*P* > 0.05). At 3 months, good/excellent MacNab rates were 96.7% in Group A vs. 88.6% in Group B (*P* = 0.396). Both groups had significant increases in all canal dimensions vs. preoperative values (*P* < 0.05). At 3 months, Group A had larger postoperative cross-sectional area and transverse diameter than Group B (*P* = 0.047 and *P* = 0.002); these represented between-group differences in postoperative values, not improvement magnitude. At 6 months, Group A had larger cross-sectional area (320.6 vs. 233.7; unadjusted *P* < 0.001, Holm-adjusted *P* = 0.002) and transverse diameter (22.7 vs. 17.3 mm, *P* < 0.001), whereas Group B's transverse diameter fell below preoperative level. Sagittal diameter showed no intergroup difference (all *P* > 0.05). Complications occurred in 1/30 (3.3%) and 2/35 (5.7%) patients (Fisher exact *P* = 1.000); revision surgery was a subsequent intervention in one of the two patients with complications.

**Conclusion:**

In this single-center retrospective cohort, both preservation and resection during AUSS were accompanied by postoperative improvement in clinical scores; short-term clinical outcomes were broadly similar, with differences only in CT-measured canal dimensions at some time points. The clinical relevance of these radiographic differences remains uncertain, and the study cannot establish equivalence, long-term efficacy, or a specific pathological mechanism.

## 1 Introduction

Degenerative lumbar spine disorders are relatively common among the elderly, with clinical manifestations primarily including low back pain, limited mobility, and intermittent claudication. The pathogenesis of these conditions is closely related to factors such as cumulative strain caused by long-term abnormal loading, decreased muscle strength resulting from insufficient physical activity, and the body's natural aging process. At present, conservative treatment is the first-line approach for these conditions; if conservative treatment proves ineffective, surgical intervention should be considered (1). The surgical approaches currently used to treat degenerative lumbar spine diseases primarily include open surgery and minimally invasive surgery; both can achieve clear therapeutic effects through decompression procedures. In comparison, open surgery is more invasive and carries a higher risk of postoperative complications; while minimally invasive surgery is less invasive, it places stricter demands on the surgeon's technical skills (2, 3).

As the most common type of degenerative lumbar spine disease, the key to surgery for lumbar disc herniation is to achieve adequate decompression while preserving the normal structure of the spine as much as possible. With continuous advances in spinal endoscopy technology, its application has gradually expanded to the treatment of degenerative lumbar spine disorders (3, 4). Compared with traditional open surgery, the AUSS (arthroscopic-assisted uni-portal spinal surgery) system offers the advantages of a small surgical incision and minimal intraoperative blood loss characteristic of minimally invasive approaches; at the same time, its non-coaxial design overcomes the spatial competition issues caused by the coaxial arrangement of surgical instruments and the endoscope in early single-port endoscopic techniques, significantly improving the surgeon's freedom of instrument manipulation within a limited operative field (2–5). It has now become one of the research hotspots in the surgical treatment of degenerative lumbar spine diseases both domestically and internationally (5).

So, should the ligamentum flavum be preserved during AUSS endoscopic surgery? Currently, there remain differing clinical perspectives on this matter. On the one hand, some scholars argue that preserving the ligamentum flavum helps maintain the integrity of the posterior wall of the spinal canal, thereby reducing the risk of postoperative epidural fibrosis and nerve root adhesions (1); on the other hand, other scholars believe that resection of the ligamentum flavum provides a more spacious operative field, facilitating the thorough removal of herniated nucleus pulposus tissue. Previous studies have addressed ligamentum flavum preservation in different settings, including an AUSS case report, a UBE comparison, and a case series, but their approaches and endpoints differed; this study provides supplementary observational data in the AUSS setting.

Based on this, we enrolled patients with lumbar disc herniation who underwent endoscopic AUSS surgery at our hospital and divided them into two groups based on whether the ligamentum flavum was preserved during surgery. The aim was to investigate the efficacy and safety of endoscopic AUSS nucleus pulposus excision with preservation of the ligamentum flavum for the treatment of lumbar disc herniation, with the hope of accumulating more clinical evidence to inform the choice of ligamentum flavum management strategies during endoscopic AUSS surgery. The findings of this study are reported below.

## 2 Materials and methods

### 2.1 General information

This study included patients with single-segment lumbar disc herniation who underwent AUSS endoscopic surgery at the Department of Spine Surgery of our hospital between September 2024 and September 2025, and conducted a retrospective analysis of their clinical data. Patients were divided into two groups based on whether the ligamentum flavum was preserved, as recorded in the surgical records:

Group A (Ligamentum flavum-preserving group): 30 patients, 17 males and 13 females, aged 20–67 years, with a mean age of (48.4 ± 12.7) years;

Group B (Ligamentum flavum-resection group): 35 patients, 16 males and 19 females, aged 22–69 years, with a mean age of (54.3 ± 13.1) years. Surgical strategy was not randomized; the decision considered interlaminar anatomy, disc location and migration, calcification, stenosis severity, and surgical level, and grouping followed the procedure actually performed. Both procedures were performed throughout the study period (September 2024 to September 2025): first half, Group A 12 and Group B 14; second half, Group A 18 and Group B 21. No attempted preservation was converted to resection (0 cases).

Inclusion Criteria:

(1) Age between 18 and 70 years;
(2) Clinically diagnosed with lumbar disc herniation, with clinical symptoms consistent with imaging findings;
(3) Lesion segment was a single-segment LDH, including L3/4, L4/5, or L5/S1;
(4) Underwent endoscopic nucleus pulposus excision (AUSS) at our hospital;
(5) Complete preoperative imaging data, including lumbar x-rays, CT, and MRI;
(6) This was the patient's first surgical treatment for a lumbar spine condition;
(7) Complete postoperative follow-up records were available, with a follow-up period of at least 6 months;
(8) Medical records were complete and included preoperative and postoperative data such as VAS scores and ODI scores.

Exclusion Criteria:

(1) Cases involving multilevel disc herniation requiring surgical intervention;
(2) Patients with concomitant lumbar spinal instability, spondylolisthesis (greater than Grade I), or isthmic fracture;
(3) Patients with severe spinal stenosis requiring extensive decompression;
(4) Patients with a history of previous lumbar surgery;
(5) Patients with concomitant lumbar infection, tumor, tuberculosis, or fracture;
(6) Conversion to open surgery or another type of endoscopic procedure during the operation;
(7) Severe osteoporosis with a T-score below −2.5;
(8) Incomplete follow-up data or loss to follow-up. The process of patient screening, exclusion, and enrollment is presented in the study flow diagram (Figure 1).

This study was approved by the Clinical Medical Research Ethics Committee of Changde First People's Hospital (approval no. 2026-453-01; reviewed on 9 August 2026 and issued on 10 August 2026). The requirement for informed consent to participate was waived, and data were de-identified before analysis.

**Figure 1:**
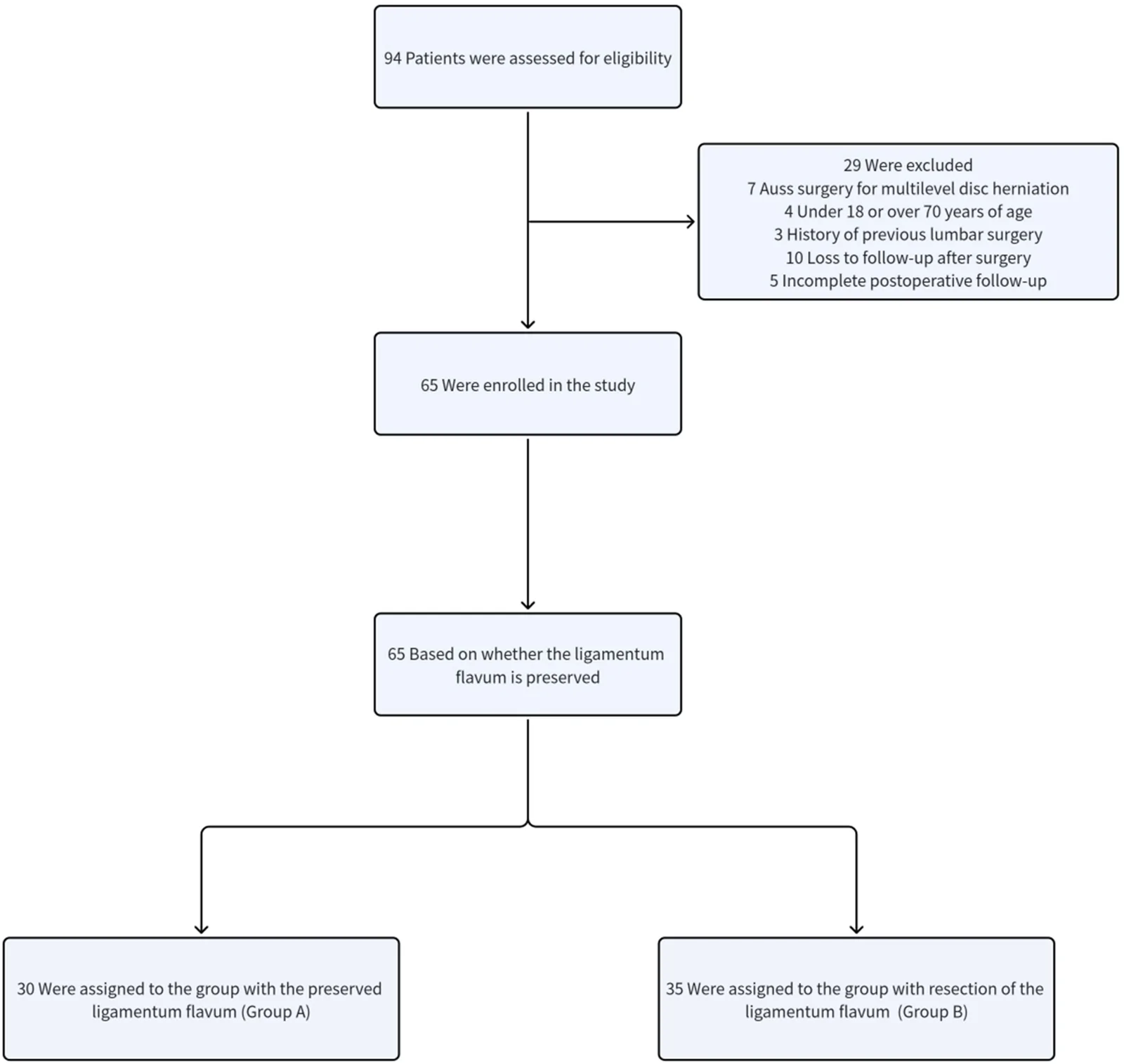
Study flow diagram.

### 2.2 Surgical procedure

Surgery was performed in both patient groups using the AUSS endoscopic system. Furthermore, all surgeries were performed by the same surgeon, who has extensive experience with AUSS. During the procedure, patients were positioned in the prone position, and the affected intervertebral disc segment was identified using a C-arm x-ray machine, followed by routine disinfection and draping. The surgical incision was made on the side of the herniated disc, approximately 1.5 cm lateral to the spinous process, as a longitudinal incision measuring 1.5–2.0 cm in length. The working channel was gradually dilated and inserted, followed by insertion of the endoscopic system.

Group A (Ligamentum flavum-preserving group): Under endoscopic visualization, the interlaminar space was exposed, and the soft tissues on the surface of the lamina were dissected to expose the superficial layer of the ligamentum flavum. Using the interlaminar approach, a working space is established between the superficial layer of the ligamentum flavum and the lamina. Free and suspend the outer edge of the ligamentum flavum and push it medially to expose the dural sac and nerve roots. Remove the herniated nucleus pulposus while preserving the integrity of the ligamentum flavum (Figure 2). Preservation was defined operationally as retention of the main body of the ligamentum flavum after release, suspension, and medial retraction of its outer attachments, with repositioning at closure; the main body was not partially or completely resected.

**Figure 2:**
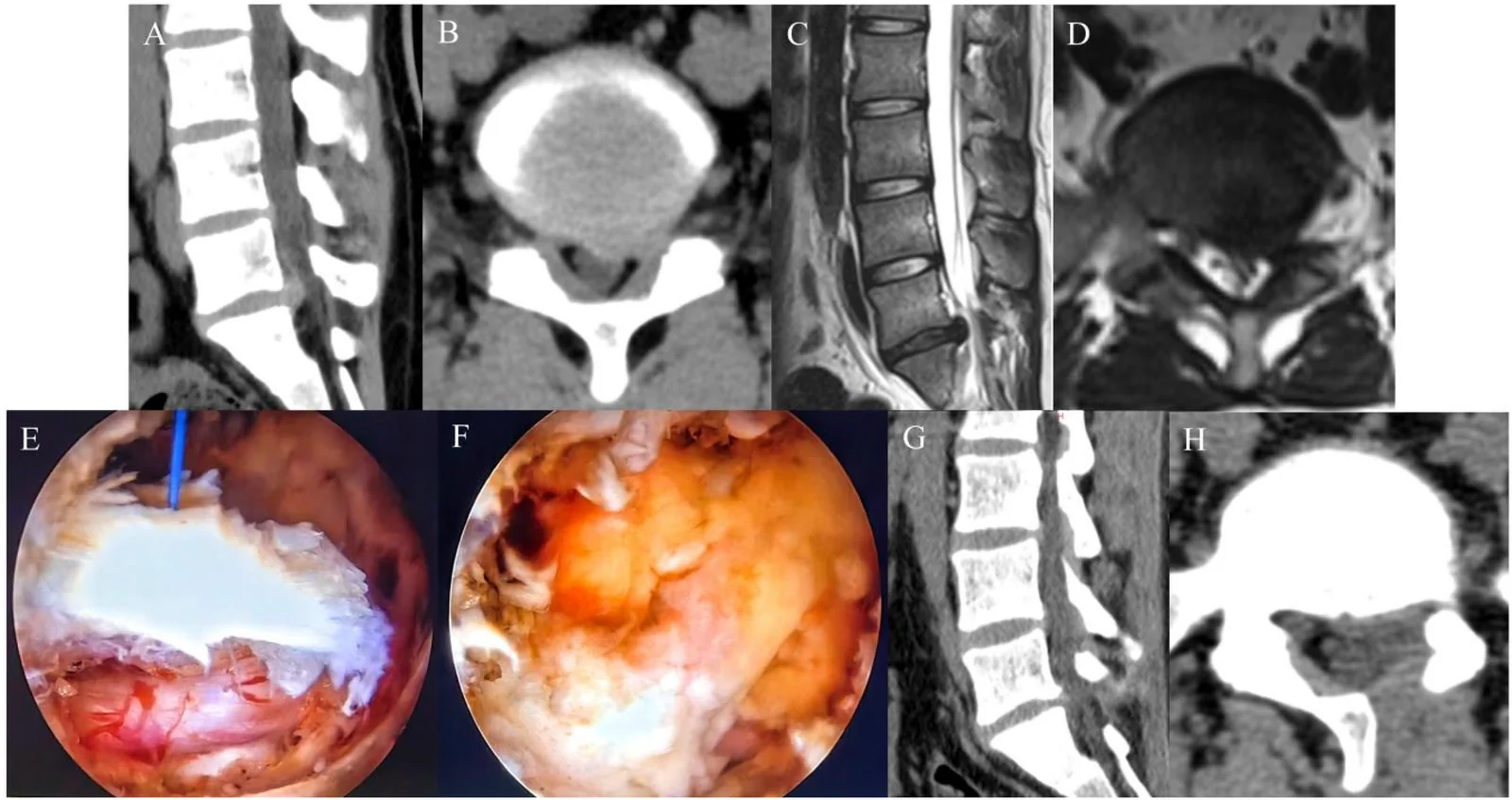
Male, 21 years old, with lumbar disc herniation at L5/S1. **(A)** Preoperative sagittal CT image shows lumbar disc herniation at L5/S1; **(B)** preoperative axial CT image shows the herniated lumbar disc compressing the nerve root and dural sac; **(C)** preoperative sagittal magnetic resonance imaging shows lumbar disc herniation at L5/S1; **(D)** preoperative axial magnetic resonance imaging shows the herniated lumbar disc compressing the nerve root and dural sac; **(E)** intraoperative image of the retained ligamentum flavum suspension; **(F)** intraoperative image of the repositioned ligamentum flavum; **(G)** postoperative sagittal CT imaging shows complete decompression of the spinal canal; **(H)** postoperative axial CT imaging shows that the herniated disc has been completely removed and the ligamentum flavum is retained.

Group B (Ligamentum Flavum Resection Group): After fully exposing the interlaminar space under endoscopic guidance, a portion of the lamina and the ligamentum flavum is first resected to expose the dural sac and nerve roots; the herniated nucleus pulposus is then removed (Figure 3). The extent of laminar or bone resection was not quantified in the operative records, and facet joint handling was not recorded.

**Figure 3:**
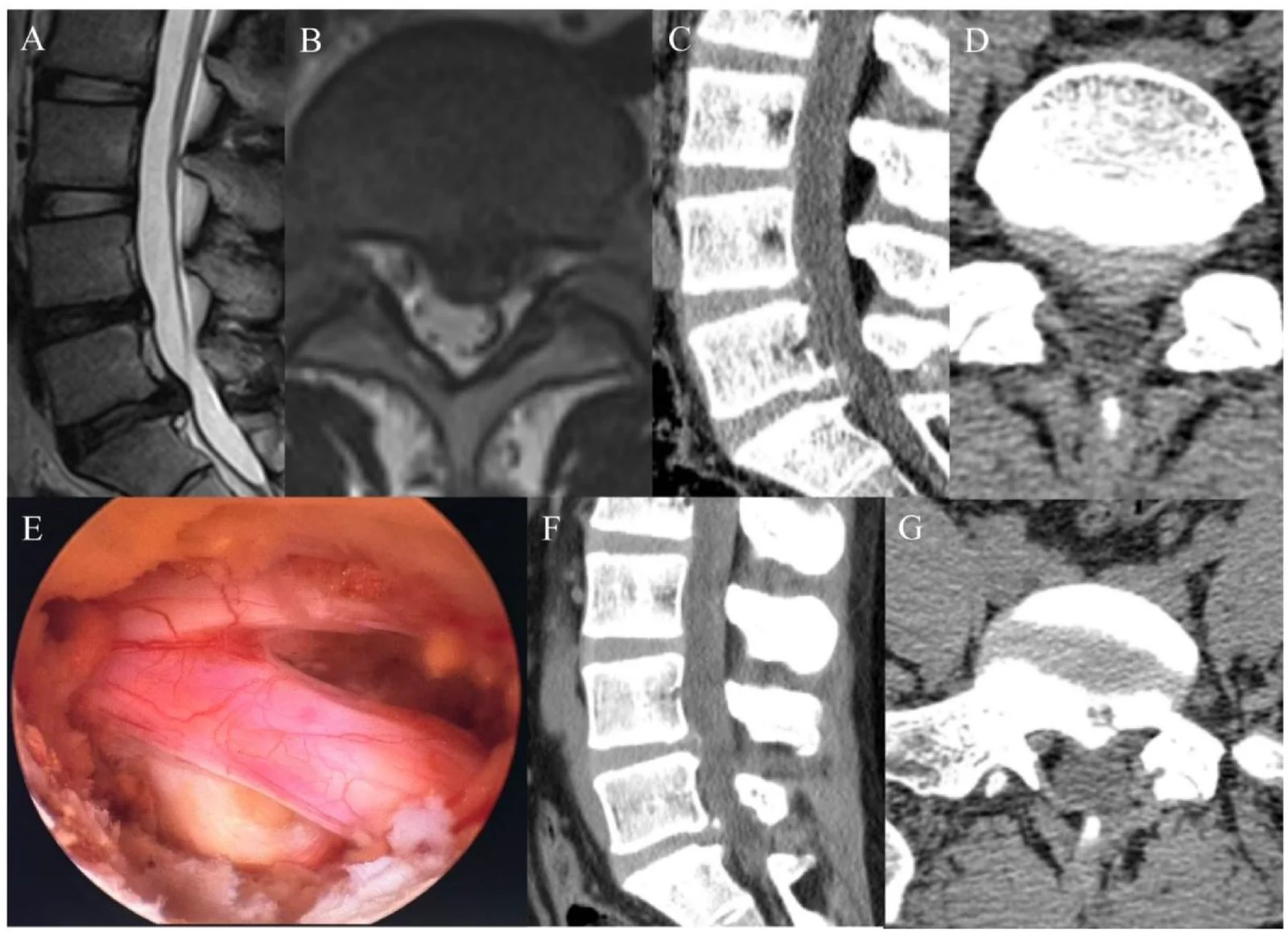
Male, 24 years old, with L5/S1 intervertebral disc herniation. **(A)** Preoperative sagittal magnetic resonance imaging shows L5/S1 intervertebral disc herniation; **(B)** preoperative axial magnetic resonance imaging shows the herniated lumbar intervertebral disc compressing the nerve root and dural sac; **(C)** preoperative sagittal CT image shows L5/S1 intervertebral disc herniation with partial calcification; **(D)** preoperative axial CT image shows the herniated lumbar intervertebral disc compressing the nerve root and dural sac; **(E)** intraoperative image after partial excision of the ligamentum flavum and complete nerve decompression; **(F)** postoperative sagittal CT image shows complete decompression of the spinal canal; **(G)** postoperative axial CT image shows that the herniated intervertebral disc has been removed. However, soft tissue hyperplasia was observed in the area where the ligamentum flavum was resected postoperatively, suggesting the possible presence of intraspinal adhesions.

Postoperatively, patients in both groups routinely received supportive treatment, including anti-infective therapy.

### 2.3 Outcome measures

#### 2.3.1 Perioperative data

The following perioperative measures were recorded: duration of surgery (minutes), intraoperative blood loss (milliliters), occurrence of intraoperative complications, length of hospital stay (days), and early postoperative complications. Observation windows: intraoperative for operative time, blood loss, and intraoperative complications; surgery day to discharge for length of stay; postoperative hospital stay for early complications; and discharge to the last follow-up (at least 6 months) for follow-up complications and revision surgery.

#### 2.3.2 Clinical efficacy assessment

##### 2.3.2.1 Pain scores

The Visual Analog Scale (VAS) was used to assess the severity of low back pain and leg pain in patients preoperatively and on the second postoperative day.

##### 2.3.2.2 Lumbar spine function assessment

Lumbar spine function was assessed using the Oswestry Disability Index (ODI) at four time points: preoperatively, on postoperative Day 2, 1 month postoperatively, and 3 months postoperatively.

##### 2.3.2.3 Efficacy evaluation

At 3 months postoperatively, treatment efficacy was evaluated using the modified MacNab criteria, with results classified into four grades: excellent, good, fair, and poor.

#### 2.3.3 Imaging parameters

CT Measurements: Using the PACS system, several imaging parameters were measured at the intervertebral disc level of the affected segment preoperatively and at 3 and 6 months postoperatively (65 patients at each time point), including the cross-sectional area of the spinal canal (unit: mm^2^), the sagittal diameter of the spinal canal (unit: mm), and the transverse diameter of the spinal canal (unit: mm). All CT-related measurements for all patients are based on the same axial slice. Measurements were made on the spinal canal (cross-sectional area, sagittal diameter, and transverse diameter) at the intervertebral disc level, using the same axial slice for all patients and time points. A formal intra- or interobserver reliability assessment was not performed. The 6-month CT data were retrieved retrospectively from existing clinical follow-up records; they were not collected prospectively as part of a preplanned protocol.

Intervertebral Space Height: On lateral x-ray images taken preoperatively and 3 months postoperatively, the heights of the anterior, middle, and posterior edges of the vertebral body were measured separately, and the average of these three measurements was calculated (unit: mm).

#### 2.3.4 Complications

The occurrence of various postoperative complications was recorded, such as incisional infection, nerve injury, recurrence, and the need for revision surgery.

### 2.4 Statistical methods

Statistical analysis of the data was performed using SPSS 26.0 software. Continuous variables that followed a normal distribution were expressed as mean ± standard deviation (x ± s) and comparisons between groups were performed using the independent samples *t*-test; continuous variables that did not follow a normal distribution were expressed as median (interquartile range), and comparisons between groups were performed using the Mann–Whitney *U*-test. Categorical data are presented as case numbers (percentages), and differences between groups were assessed using the *χ*^2^ test or Fisher's exact test. No baseline-adjusted ANCOVA or mixed-effects model was performed, and no level-stratified sensitivity analysis was performed; therefore, the results are based on unadjusted between-group comparisons. For the six CT comparisons (three parameters at two postoperative time points), the reported two-sample *t*-test *P* values were corrected for multiplicity using the Holm method, and raw and adjusted *P* values are reported. A *P*-value < 0.05 was considered statistically significant. No primary outcome or primary time point was prespecified, and all comparisons are exploratory. Paired change-from-baseline confidence intervals were not calculated.

## 3 Results

### 3.1 Comparison of baseline characteristics between the two groups

The groups were similar in sex, BMI, herniation severity, preoperative comorbidity, and perioperative parameters, but differed in age (48.4 ± 12.7 vs. 54.3 ± 13.1 years), surgical level (L4/5: 11 vs. 23; L5/S1: 19 vs. 11; L3/4: 0 vs. 1), herniation type (central: 15 vs. 25; paracentral: 3 vs. 0; lateral: 12 vs. 10), and preoperative VAS (*P* = 0.001). Standardized mean differences were 0.46 (age), 0.70 (level), 0.60 (herniation type), and 0.89 (preoperative VAS); non-significant *P* values were not used to establish baseline comparability. No adjusted analysis was performed. No complications, such as nerve root injury or dural sac rupture, occurred in either group during surgery. One patient in Group B developed a cerebrospinal fluid leak postoperatively, which resolved after conservative treatment (Table 1) (Figure 4).

**Table 1:** Comparison of baseline and perioperative data between two groups.

Characteristic		Group A (*n* = 30)	Group B (*n* = 35)	t/χ²	*P*
Male/female		17/13	16/19	0.466	0.495
Age (years)		48.4 ± 12.7	54.3 ± 13.1	1.964	0.054
BMI (kg/m^2^)		24.8 ± 3.5	25.0 ± 4.0	0.215	0.831
Disc herniation classiﬁcation	Bulging	2	2	0.097	0.953
	Protrusion	26	31		
	Extrusion	2	2		
Surgical segment	L3/4	0	1	5.802	0.055
	L4/5	11	23		
	L5/S1	19	11		
Herniation type	Central	15	25	4.804	0.090
	Paracentral	3	0		
	Lateral	12	10		
Preoperative comorbidity		10 (33.3%)	13 (37.1%)	0.092	0.762
Hospitalization time (days)		14.5 ± 6.8	14.9 ± 5.4	0.258	0.797
Operation time (min)		146.3 ± 42.1	156.9 ± 59.8	0.822	0.414
Intraoperative blood loss (mL)		32.5 ± 23.1	34.5 ± 36.2	0.257	0.798
Intraoperative Complication		0 (0%)	0 (0%)	–	–
Early postoperative complications		1 (3.3%)	2 (5.7%)	–	1.000

Numeric data were expressed as Mean ± SD and analyzed by Independent-Samples *T*-test. Categorical data were expressed by the number of patients (%) and were analyzed with the *χ*^2^ test. Group A: Group with preserved ligamentum flavum; Group B: Group with resected ligamentum flavum. BMI, body mass index; AUSS, arthroscopic-assisted uni-portal spinal surgery.

**Figure 4:**
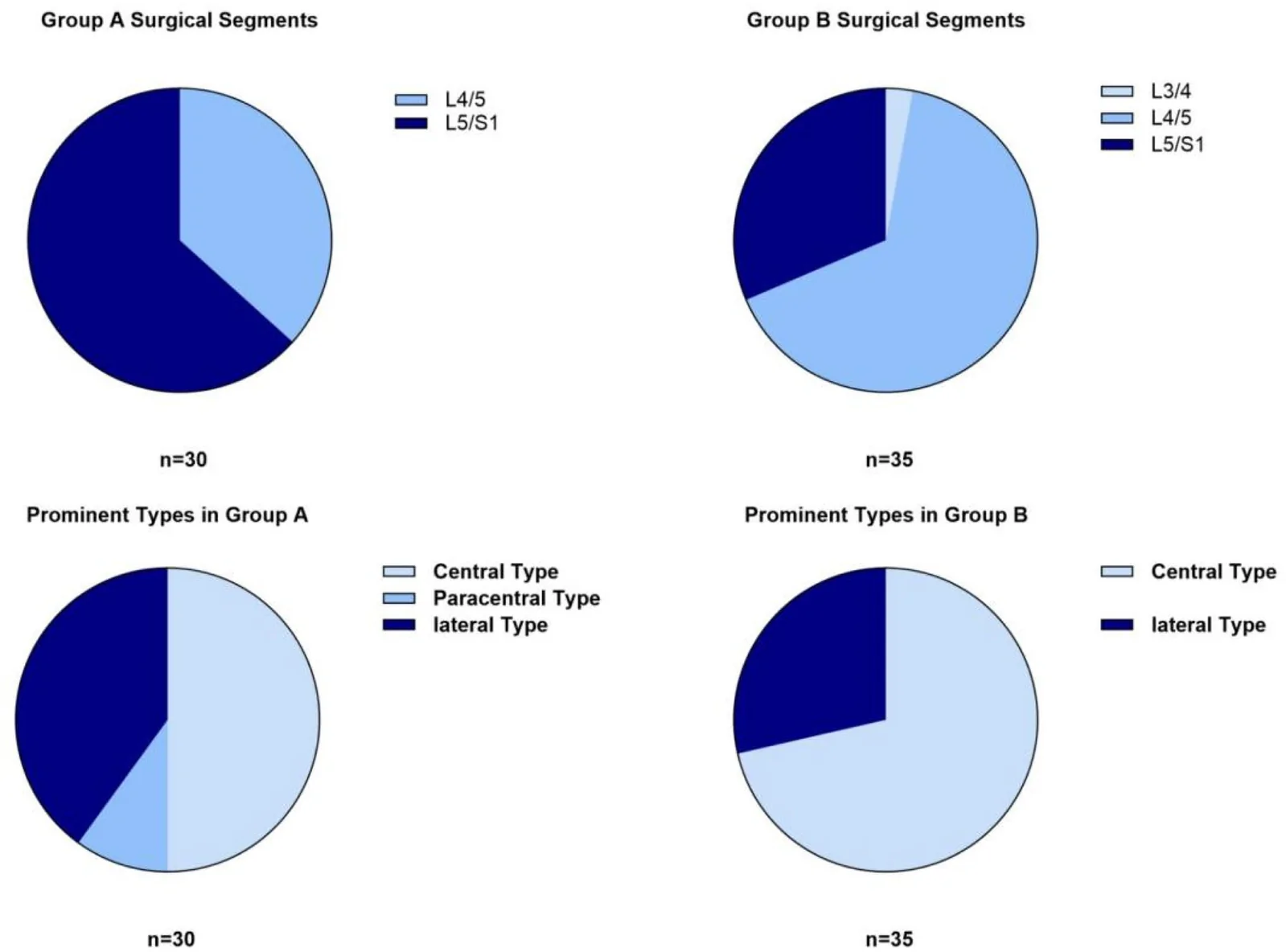
This figure shows that surgeries in Group A primarily involved the L5/S1 segment, with a diverse range of herniation types; surgeries in Group B were concentrated in the L4/5 segment, with the vast majority being central disc herniations, only a small number of lateral herniations, and very few cases involving the higher L3/4 segment. Group A: Group with preserved ligamentum flavum; Group B: Group with resected ligamentum flavum.

### 3.2 Comparison of clinical efficacy between the two groups

There was a statistically significant difference in preoperative VAS scores for low back pain and leg pain between the two groups (*P* < 0.05). On the second postoperative day, VAS scores in both groups decreased significantly compared to preoperative levels (*P* < 0.05), but there was no statistically significant difference between the groups (*P* > 0.05). There was no statistically significant difference in preoperative ODI scores between the two groups (*P* > 0.05). At all postoperative time points, ODI scores in both groups decreased significantly compared to preoperative levels (*P* < 0.05), but there was no statistically significant difference between the groups (*P* > 0.05). At 3 months postoperatively, the rate of “good” or “excellent” outcomes was 96.7% (29/30) in Group A and 88.6% (31/35) in Group B; the difference between the two groups was not statistically significant (*Z* = 0.849, *P* = 0.396) (Table 2) (Figure 5). No adjusted between-group estimates were computed; only unadjusted comparisons are reported.

**Table 2:** Comparison of pain VAS, ODI scores and modified MacNab criteria at preoperative and postoperative time points.

Outcome	Time point	Group A (*n* = 30)	Group B (*n* = 35)	Statistical value	*P*
Low back pain VAS	Pre-operation	6.5 ± 0.9	7.3 ± 0.9	3.571	0.001
	Post-operation (2 days)	1.1 ± 0.3*	1.4 ± 1.0*	1.524	0.133
Leg pain VAS	Pre-operation	6.4 ± 0.9	7.2 ± 0.9	3.540	0.001
	Post-operation (2 days)	1.1 ± 0.3*	1.4 ± 0.9*	1.656	0.103
ODI	Pre-operation	51.8 ± 4.1	52.4 ± 2.8	0.693	0.491
	Post-operation (2 days)	22.9 ± 2.5*	23.4 ± 3.3*	0.677	0.501
	Post-operation (1 month)	13.1 ± 2.7*	15.0 ± 6.4*	1.448	0.153
	Post-operation (3 months)	9.1 ± 2.0*	9.6 ± 4.9*	0.512	0.611
Modified MacNab	Excellent	1 (3.3%)	1 (2.9%)	–	–
	Good	28 (93.3%)	30 (85.7%)	–	–
	Fair	1 (3.3%)	3 (8.6%)	–	–
	Poor	0 (0%)	1 (2.9%)	–	–
	Excellent/good rate	29 (96.7%)	31 (88.6%)	0.849†	0.396†

Numeric data were expressed as Mean ± SD and analyzed by Independent-Samples *T*-test. *P < 0.05 versus the preoperative value within the same group. Categorical data were expressed by the number of patients (%) and were analyzed with the χ² test. ^†^Mann-Whitney U test. Group A: Group with preserved ligamentum flavum; Group B: Group with resected ligamentum flavum. VAS, visual analog scale, ODI, oswestry disability index, AUSS, arthroscopic-assisted uni-portal spinal surgery.

**Figure 5:**
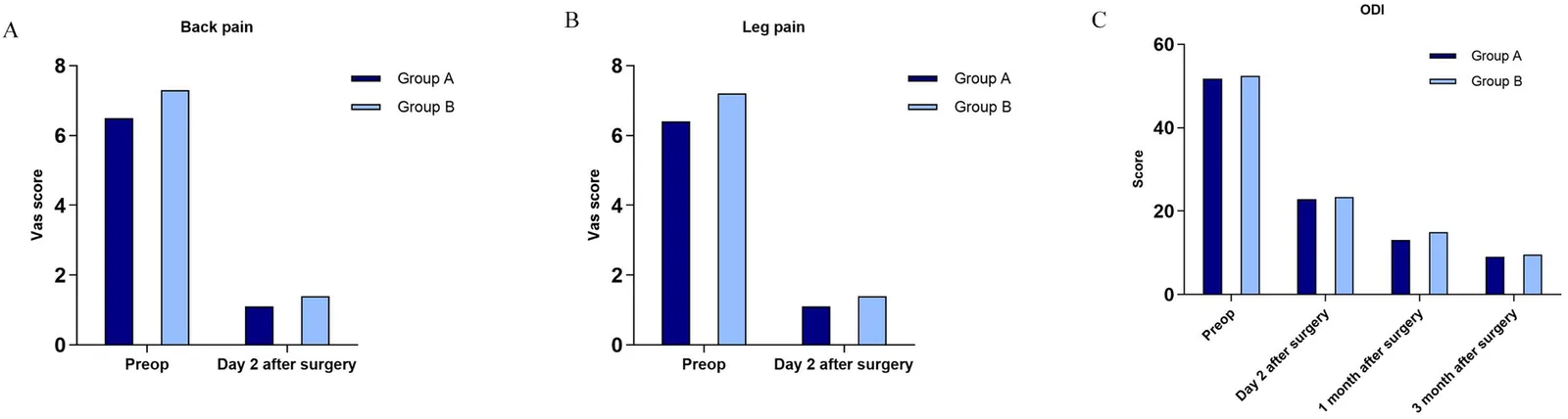
This figure provides a comprehensive comparison of the VAS scores for low back pain and leg pain, as well as ODI (Oswestry Disability Index) scores, for patients in Groups A and B at multiple time points before and after surgery. Regarding low back pain, both groups had high preoperative VAS scores (Group A: 6.5; Group B: 7.3), which decreased significantly by Day 2 postoperatively (Group A: 1.1; Group B: 1.4), with similar degrees of pain relief in both groups. Regarding leg pain, patients in both groups experienced severe leg pain preoperatively (Group A: 6.4 points; Group B: 7.2 points), and scores decreased markedly 2 days postoperatively (Group A: 1.1 points; Group B: 1.4 points), with a similar degree of improvement in leg pain between the two groups. Regarding functional impairment, the preoperative ODI scores were similar between the two groups; as the postoperative recovery period progressed, ODI scores in both groups continued to decline, and the differences in scores between the two groups were minimal at all follow-up time points. Overall, patients in both groups experienced significant pain relief in the early postoperative period, and the trends in functional recovery were consistent, with no significant differences between the groups. **(A)** VAS score for low back pain; **(B)** VAS score for leg pain; **(C)** ODI score.

### 3.3 Comparison of radiological parameters between the two groups of patients

#### 3.3.1 Spinal canal morphological parameters

At 3 months postoperatively, the cross-sectional area, sagittal diameter, and transverse diameter of the spinal canal in both groups had significantly increased compared with preoperative measurements (*P* < 0.05). In the intergroup comparison, the postoperative cross-sectional area of the spinal canal was larger in Group A than in Group B (*P* = 0.047), which is a between-group difference in postoperative values rather than in the magnitude of improvement. At 6 months postoperatively, the spinal canal cross-sectional area was larger in Group A than in Group B (320.6 vs. 233.7; unadjusted *P* < 0.001, Holm-adjusted *P* = 0.002). For the sagittal diameter of the spinal canal, there were no significant differences between the two groups at any of the three time points (all *P* > 0.05);for the transverse diameter of the spinal canal, the difference was most significant at 6 months postoperatively (*t* = 4.467, *P* < 0.001) (Table 3) (Figure 6). CT measurements were available for all 65 patients at each time point (65/65/65). Holm-adjusted *P* values for the six CT comparisons were: cross-sectional area 0.140 (3 months) and 0.002 (6 months); sagittal diameter 0.678 (3 months) and 0.303 (6 months); transverse diameter 0.006 (3 months) and <0.001 (6 months). After Holm correction, the 3-month cross-sectional area difference was no longer statistically significant. No baseline-adjusted estimates were computed.

**Table 3:** Imaging morphological parameters at preoperative and postoperative follow-up at 3 and 6 months.

Parameter	Time point	Group A (*n* = 30)	Group B (*n* = 35)	t	P
Cross-sectional area (mm^2^)	Pre-operation	232.7 ± 83.2	227.8 ± 73.5	0.263	0.794
	Post-operation (3 months)	322.5 ± 94.8	275.1 ± 94.9	2.028	0.047
	Post-operation (6 months)	320.6 ± 79.8	233.7 ± 109.5	3.687	<0.001
Sagittal diameter (mm)	Pre-operation	15.5 ± 3.8	16.4 ± 3.6	1.018	0.313
	Post-operation (3 months)	16.9 ± 3.6	17.3 ± 4.3	0.417	0.678
	Post-operation (6 months)	16.8 ± 2.6	15.6 ± 4.0	1.451	0.152
Transverse diameter (mm)	Pre-operation	18.5 ± 4.5	18.0 ± 4.6	0.438	0.663
	Post-operation (3 months)	23.6 ± 4.4	19.8 ± 4.6	3.304	0.002
	Post-operation (6 months)	22.7 ± 4.3	17.3 ± 5.4	4.467	<0.001

Numeric data were expressed as Mean ± SD and analyzed by Independent-Samples *T*-test. CT measurements were available for 65/65/65 patients; Holm-adjusted *P* values are reported in the Results. Baseline-adjusted estimates were not computed.

**Figure 6:**
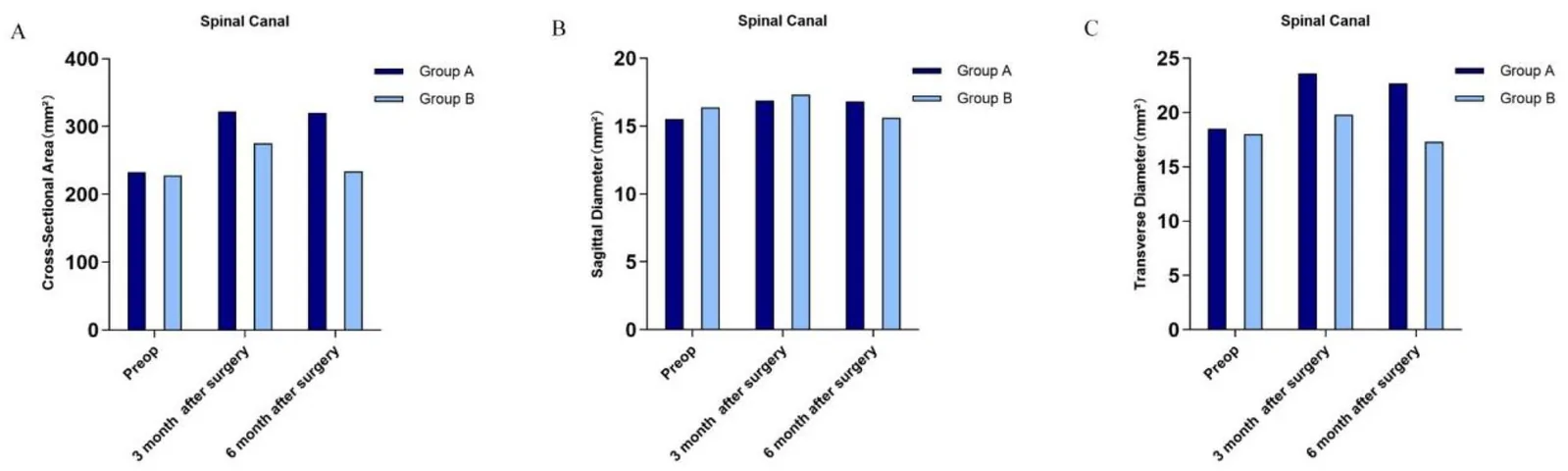
This figure presents a comprehensive comparison of the morphological parameters of the spinal canal in patients from Group A and Group B at preoperative and 3-month and 6-month postoperative time points, covering three dimensional indicators: cross-sectional area, sagittal diameter, and transverse diameter. Regarding the cross-sectional area of the spinal canal, the preoperative values were similar between the two groups. Both groups showed an increasing trend at 3 months postoperatively, followed by a decreasing trend at 6 months postoperatively compared to 3 months. At all follow-up time points, the cross-sectional area in Group A was greater than that in Group B, and both groups exhibited the most significant degree of expansion at 3 months postoperatively. Regarding the sagittal diameter of the spinal canal, both groups showed a significant increase at 3 months postoperatively; at 3 months postoperatively, Group B had higher values; however, at 6 months postoperatively, Group A demonstrated better improvement than Group B. For the transverse diameter of the spinal canal, the preoperative values of both groups were also similar, and both showed a significant increase at 3 months postoperatively; by 6 months postoperatively, Group A demonstrated better improvement than Group B. At all follow-up time points, the transverse diameter in Group A was greater than that in Group B. Overall, the spinal canal morphology in both groups showed significant improvement at 3 months postoperatively; however, by 6 months postoperatively, the morphology had regressed somewhat compared to the 3-month mark, with a more pronounced regression in Group B than in Group A. **(A)** Cross-sectional area; **(B)** Sagittal diameter; **(C)** Transverse diameter.

#### 3.3.2 Intervertebral space height

A comparison of intervertebral disc height between the two groups before surgery and 3 months after surgery showed no statistically significant differences (*P* > 0.05) (Table 4).

**Table 4:** Intervertebral disc height at preoperative and 3-month postoperative time points.

Parameter	Time point	Group A (*n* = 30)	Group B (*n* = 35)	*t*	*P*
Disc height (mm)	Pre-operation	8.8 ± 4.0	8.6 ± 2.8	0.225	0.822
	Post-operation (3 months)	8.8 ± 2.6	8.5 ± 2.4	0.479	0.634

Values are expressed as the mean ± standard deviation.

### 3.4 Complications

One case of postoperative infection occurred in Group A (3.3%), while Group B had one case of incisional hematoma complicated by vertebral infection (2.9%), one case of cerebrospinal fluid leak (2.9%). Complications occurred in 1/30 (3.3%) patients in Group A and 2/35 (5.7%) in Group B. The revision surgery was a subsequent intervention in one of the two Group B patients (1/35), not a third complication. The two-sided Fisher exact test gave *P* = 1.000; the Clopper-Pearson 95% CIs were 0.08% to 17.22% and 0.70% to 19.16%, and the risk difference (A-B) was −2.38 percentage points (Newcombe 95% CI −15.56 to +11.58). There were no cases of nerve injury or recurrence in either group.

## 4 Discussion

With an aging population, degenerative lumbar spine disease has become a common condition among the elderly. Currently, treatment options for this condition primarily include conservative management and surgical intervention, with conservative management being the first-line approach. If conservative management fails to produce satisfactory results, surgical intervention should be considered. Surgical approaches can be further categorized into open surgery and minimally invasive surgery. In recent years, the use of endoscopes in minimally invasive spinal surgery has been on the rise, although it requires a high level of skill and technical proficiency. Endoscopy is gradually becoming an integral part of modern spinal surgery (4, 5). Compared with open and traditional minimally invasive procedures, endoscopic spinal surgery offers better functional outcomes, faster recovery, earlier initiation of functional exercises, and a reduction in complications associated with prolonged postoperative bed rest (2, 3, 5).

This study observed and documented the clinical outcomes of the ligamentum flavum preservation group and the ligamentum flavum resection group, respectively, with the aim of better comparing the differences in clinical efficacy for the treatment of lumbar disc herniation when performing endoscopic AUSS with or without ligamentum flavum preservation. Preliminary results indicate that, during the follow-up period, postoperative CT-measured cross-sectional area of the spinal canal was larger in the preservation group at some time points; because allocation was non-random and the groups differed in surgical level and herniation type, this finding is hypothesis-generating and is compatible with, but does not establish, a causal effect of preservation. Apart from this, differences in other clinical outcomes were not statistically significant.

With continuous advances in imaging and diagnostic and therapeutic technologies, coupled with the growing number of patients with spinal disorders due to an aging population, a wide variety of minimally invasive surgical techniques have emerged (3). Overall, minimally invasive surgery has two primary goals: first, to enable patients to recover sooner and begin functional exercises earlier; and second, to achieve decompression as thorough as that of open surgery (2, 4, 5). The clinical value of preserving the ligamentum flavum has been thoroughly confirmed by numerous studies. Its core benefits extend across surgical safety, the postoperative rehabilitation process, and long-term prognosis, and it has become a key strategy in modern minimally invasive lumbar surgery.

The ligamentum flavum is a normal physiological structure in the human body, rich in elastic fibers, which account for 70% to 80% of its composition. These fibers are arranged in parallel longitudinal rows, interwoven with a network of collagen fibers, giving the ligamentum flavum both high elasticity and strong tear resistance. Anatomically, it lies in close proximity to the dural sac, the vertebral lamina, and the articular joint capsule, thereby laying the foundation for the dynamic stability of the spine and the protection of nerves.

During surgery, preserving the ligamentum flavum intact allows it to fulfill its core function as a natural anatomical barrier within the spinal canal. Previous literature has proposed that preservation may reduce postoperative scar tissue and maintain mechanical stability (6); however, this study did not directly measure fibrosis. These proposed barrier functions have been described in previous studies but were not directly assessed in the present study. Previous controlled studies have reported less epidural fibrosis after preservation; however, these findings were not verified in the present study, which did not directly assess fibrosis. Preservation has been proposed to reduce the risk of postoperative pain and neurological dysfunction; however, clinical outcomes did not differ significantly between groups in this study. Some postoperative radiographic parameters of the spinal canal were larger in the preservation group at selected time points, suggesting that preservation may help maintain the postoperative anatomy of the spinal canal. Preservation has also been proposed to improve intraoperative safety and reduce dural tears or cerebrospinal fluid leaks; however, complication rates did not differ significantly between groups in this study. This proposed protective effect has been described in prior studies (6); however, the present study did not demonstrate significant differences in complication rates. Moreover, this technique does not increase surgical difficulty or prolong operative time, making it highly feasible in patients with uncomplicated lumbar disc herniation. Long-term studies from other groups have reported better outcomes with preservation (7); however, the present study was limited to 6 months and cannot confirm long-term prognostic benefits. Preservation may also facilitate future revision surgery (6), although this was not assessed in the present study. Combining preservation with annular repair has been proposed to maintain posterior ligamentous integrity and reduce recurrence (6); however, this combination was not evaluated in the present study. These potential advantages remain to be verified in prospective studies. Furthermore, the ligamentum flavum can serve as an anatomical landmark during surgical procedures and subsequent revision surgeries, helping surgeons locate the target area more quickly. Because this study did not directly measure epidural fibrosis, segmental stability, or revision-related outcomes, these potential mechanisms and long-term benefits require further evaluation in prospective studies.

Although intraoperative resection of the ligamentum flavum facilitates better exposure of the surgical site, this procedure may alter the posterior structures of the spinal canal and the local biomechanical environment. First, the ligamentum flavum is a crucial elastic stabilizing structure within the posterior column of the spine. Its loss may theoretically affect segmental stability and increase the risk of abnormal motion, degeneration, and postoperative segmental instability (7, 8). Previous studies have proposed that the fatty septum may limit scar tissue invasion and that resection may increase the risk of epidural fibrosis and adhesions (9); however, fibrosis was not directly assessed in the present study. At the same time, the smooth inner surface of the ligamentum flavum and its dynamic elastic recoil properties protect the dural sac and nerve roots during spinal motion, preventing them from being subjected to abnormal friction and compression. After resection of the ligamentum flavum, this cushioning barrier is lost, increasing the risk of irritation or even injury to the spinal cord and nerve roots during movement. Loss of the ligamentum flavum may reduce the canal's ability to adjust its volume, and postoperative soft-tissue changes may contribute to a secondary reduction in canal volume (10); however, this mechanism was not directly assessed. Combined with postoperative scar tissue contracture, some patients may experience a secondary reduction in effective spinal canal volume, which may even exacerbate symptoms of nerve compression (10). Previous controlled studies have reported less favorable outcomes after resection (11); however, the present study did not confirm significant differences in pain, function, or reoperation. In specific conditions such as ossification of the ligamentum flavum, resection has been reported to increase the risk of dural tears and cerebrospinal fluid leaks (9, 12); complication rates did not differ significantly in this cohort. In our study, the group in which the ligamentum flavum was preserved did not experience a significant increase in surgical difficulty or operating time, a finding consistent with previous research (13), and intraoperative blood loss was numerically lower than in the resection group, although this difference was not statistically significant. Analysis suggests this may be related to the fact that the resection group required additional dissection and traction of the soft tissues on the posterior wall of the spinal canal, thereby exacerbating local vascular injury (12, 13). During the available short-term follow-up, endoscopic discectomy performed via the AUSS approach—regardless of whether the ligamentum flavum was preserved or resected—was associated with improvement in lower back and leg pain and lumbar function; these results were comparable to those of traditional methods (13). Furthermore, neither group exhibited a significant postoperative decrease in intervertebral disc height, indicating that both surgical approaches have a minimal impact on intervertebral disc stability. At three months postoperatively, the postoperative cross-sectional area was larger in Group A than in Group B (322.5 ± 94.8 mm^2^ vs. 275.1 ± 94.9 mm^2^; unadjusted *P* = 0.047). This represents an unadjusted between-group comparison of postoperative values, not a difference in the magnitude of improvement. After Holm correction, the difference was not statistically significant (adjusted *P* = 0.140). The larger postoperative cross-sectional area in Group A may be compatible with differences in postoperative soft-tissue changes; however, epidural fibrosis was not directly assessed, and this finding cannot establish that ligamentum flavum preservation prevents scar tissue invasion. Imaging at 6 months postoperatively showed that the spinal canal cross-sectional area was larger in Group A than in Group B (320.6 vs. 233.7; unadjusted *P* < 0.001, Holm-adjusted *P* = 0.002). In Group B, the area decreased from 275.1 at 3 months postoperatively to 233.7, These CT changes are compatible with postoperative soft-tissue changes, but fibrosis was not directly assessed; slice matching, measurement variability, bony decompression, and other anatomical changes may also contribute.Sagittal diameter of the spinal canal: No significant differences were observed between the two groups at any of the three time points (all *P* > 0.05), suggesting that the sagittal diameter may not be a sensitive indicator for evaluating the value of preserving the ligamentum flavum. Transverse diameter of the spinal canal: The difference was most significant at 6 months postoperatively (*t* = 4.467, *P* < 0.001). Group A maintained good expansion (22.7 mm), while Group B's measurement was even lower than the preoperative level (17.3 vs. 18.0 preoperatively), which may be compatible with a role of postoperative soft-tissue changes, although fibrosis was not directly assessed. Regarding the MacNab score: The rate of excellent or good outcomes in the group with preserved ligamentum flavum was 96.7% (29/30), while in the group with resected ligamentum flavum, it was 88.6% (31/35). In terms of complications, the incidence rate in Group A (ligamentum flavum preservation group) was 3.3% (1/30), while in Group B (ligamentum flavum resection group) it was 5.7% (2/35). One case of postoperative infection occurred in Group A. In Group B, one patient developed an incisional hematoma complicated by vertebral infection and subsequently underwent revision surgery, and one patient developed a cerebrospinal fluid leak; therefore, the revision surgery was a subsequent intervention in one of the two patients with complications, not an additional third complication. These observations are presented alongside prior studies; our study was not designed to test whether resection increases these risks. The present data cannot establish a causal link between ligamentum flavum resection and revision surgery. Adhesions pose multiple problems: not only do they cause persistent postoperative low back pain and recurrence of neurological symptoms, but they also obscure anatomical landmarks during revision surgery, making dissection difficult. This significantly increases the complexity of the procedure and the risk of nerve injury, ultimately leading to a marked rise in the revision rate. As for postoperative infection, it may be related to the following factors: a relatively large extent of soft tissue dissection, prolonged operative time, and increased intraoperative blood loss. All of these factors raise the probability of contamination in the surgical site, thereby creating conditions conducive to bacterial colonization. Once an infection occurs, it often requires long-term antimicrobial therapy and debridement, which undoubtedly places a significant burden on the healthcare system and causes additional suffering for the patient (14, 15). In our study, patients who developed complications showed improvement following conservative treatment or revision surgery. Therefore, establishing a rigorous clinical strategy prior to surgery, standardizing surgical procedures, and implementing scientific, comprehensive, and individualized postoperative care are crucial for preventing such complications (14, 15). In summary, resection of the ligamentum flavum is not without potential risks; biomechanical changes, scar adhesions, and loss of neural protection should be considered in clinical decision-making (12). This study has certain limitations that need to be addressed in clinical practice and future research. First, as this was a single-center retrospective study, the generalizability of the results may be limited; furthermore, the small sample size (30 patients in Group A and 35 in Group B) may introduce patient selection bias. Second, the follow-up period was only 6 months; further observation is needed to assess long-term postoperative outcomes, including spinal degeneration, disc reherniation, changes in adjacent vertebrae, and radiological changes. Third, due to patients’ financial circumstances and personal preferences, we were unable to routinely perform MRIs during follow-up to assess epidural fibrosis; finally, this study focused solely on patients with simple lumbar disc herniation. For patients with complex conditions such as ligamentum flavum hypertrophy, lumbar spinal stenosis, or mild lumbar instability, the feasibility, surgical techniques, and efficacy of ligamentum flavum preservation remain unclear. Future studies could conduct subgroup analyses stratified by pathological type and age to further refine the indications and contraindications for ligamentum flavum preservation and develop individualized surgical plans. Therefore, the impact of ligamentum flavum preservation on postoperative scar formation requires further investigation. In the future, we need to prioritize conducting multicenter, prospective, large-sample randomized controlled trials to further validate and expand the long-term efficacy of nucleus pulposus excision with preservation of the ligamentum flavum. In addition, this was a single-center, single-surgeon, small-sample retrospective study; the findings apply only to the population and setting studied and cannot be directly extrapolated to other centers, surgeons in the learning curve, or excluded populations such as patients with severe stenosis, previous lumbar surgery, or multilevel disease. Multicenter prospective validation is needed. Because baseline data for patients excluded due to follow-up issues were largely unavailable, an included-vs.-excluded comparison could not be performed, and the direction of attrition-related bias is uncertain. Because this study did not directly evaluate the degree of fibrosis, dynamic stability, or changes in canal volume and was limited by a small sample size and short follow-up, the long-term effects of ligamentum flavum resection require validation in long-term prospective studies.

## 5 Conclusion

In this single-center retrospective cohort, both preservation and resection of the ligamentum flavum during AUSS were accompanied by postoperative improvement in clinical scores. Short-term clinical outcomes were broadly similar between groups, and differences were observed only in CT-measured canal dimensions at some time points. The clinical relevance of these radiographic differences remains uncertain; the study cannot establish equivalence or non-inferiority, nor can it support long-term efficacy or specific pathological mechanisms.

## Data Availability

The original contributions presented in the study are included in the article/Supplementary Material, further inquiries can be directed to the corresponding author/s.
